# Supplementary figures and images for: Sex Differences in a Novel Mouse Model of Spinocerebellar Ataxia Type 1 (SCA1)
Source: Int J Mol Sci. 2025 Mar 14;26(6):2623. doi: 10.3390/ijms26062623 (PMC11942590; doi:10.3390/ijms26062623)

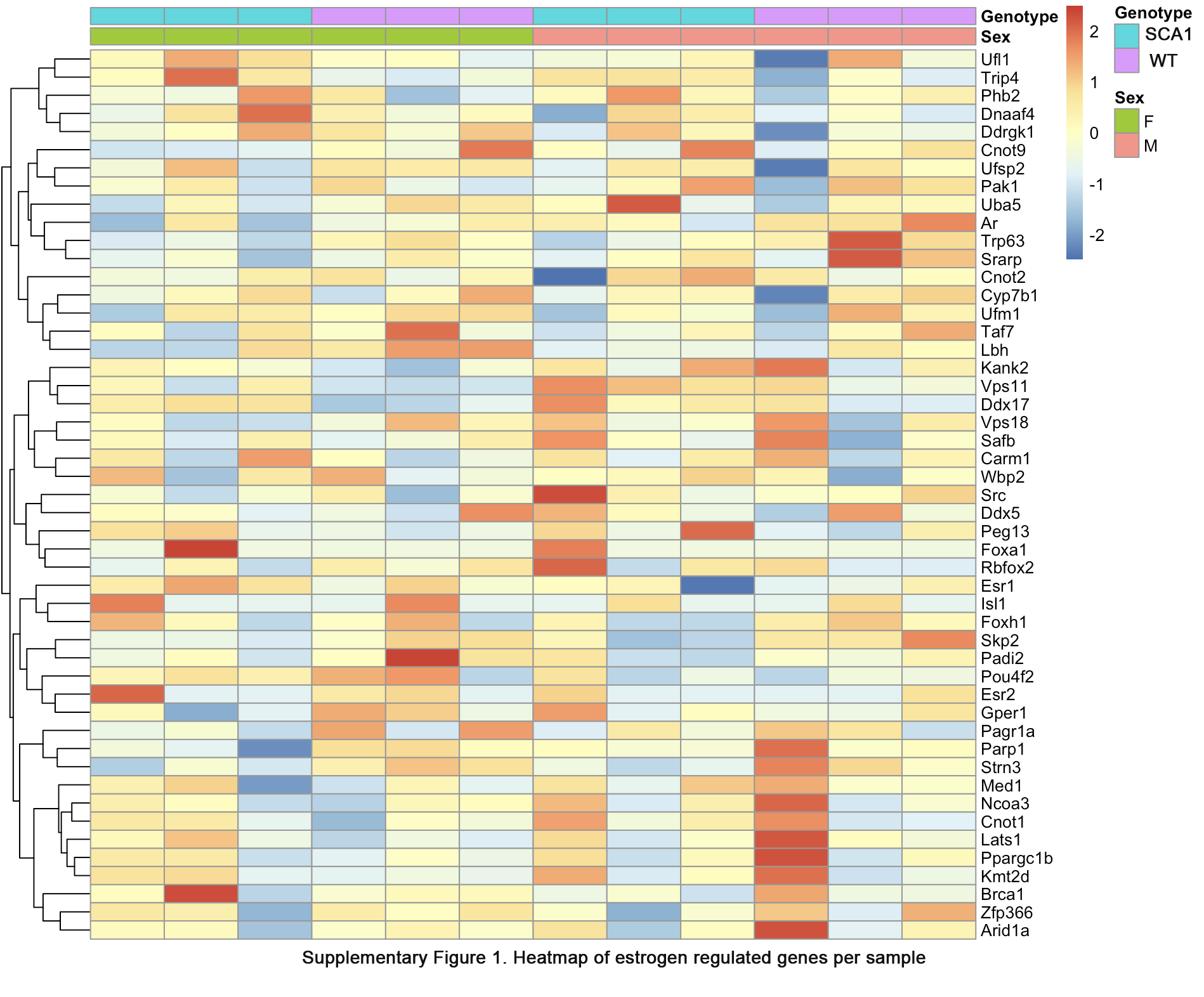

Supplement: Supplementary file 1 [file ijms-26-02623-s001.zip › ijms-3511291-Figure S1.tif]
